# Syncope as the Initial Manifestation of Advanced Nasopharyngeal Carcinoma: A Case Report

**DOI:** 10.3389/fcvm.2021.796653

**Published:** 2022-01-10

**Authors:** Si-Cheng Zhang, Mao-Qing Lin, Li-Wei Zhang, Xue-Qin Lin, Man-Qing Luo, Kai-Yang Lin, Yan-Song Guo

**Affiliations:** ^1^Department of Cardiology, Shengli Clinical Medical College of Fujian Medical University, Fujian Provincial Hospital, Fuzhou, China; ^2^Fujian Provincial Key Laboratory of Cardiovascular Disease, Fujian Cardiovascular Institute, Fujian Provincial Center for Geriatrics, Fujian Provincial Clinical Research Center for Severe Acute Cardiovascular Diseases, Fuzhou, China

**Keywords:** syncope, nasopharyngeal carcinoma, carotid sinus syndrome, diagnosis, case report

## Abstract

Carotid sinus syndrome is a principal cause of syncope in the elderly. Syncope, associated with carotid sinus syndrome which is secondary to metastasis of advanced nasopharyngeal carcinoma, rarely occurs. The current study reported a 66-year-old woman, who presented with a history of frequent and recurrent syncope as the initial symptom, and was eventually diagnosed with advanced nasopharyngeal carcinoma. The positron emission tomography scan demonstrated a diagnosis of advanced nasopharyngeal carcinoma with involvement in carotid sheath space, and nasopharyngeal biopsy revealed non-keratinized nasopharyngeal carcinoma. After diagnosis and treatment, the patient had no recurrence of syncope. In summary, our case study suggests that great importance should be attached to potential intrinsic causes of syncope especially in the case of nasopharyngeal carcinoma, as it is an insidious malignancy which needs to be precisely identified.

## Introduction

Carotid sinus syndrome is a principal cause of syncope among the elderly, accounting for 26 to 60% of elderly patients with inexplicable syncope (1, 2). Patients with advanced nasopharyngeal carcinoma usually present with blood-tinged sputum, nosebleed, hearing loss, headache and neck mass. Nevertheless, syncope, associated with the metastasis of advanced nasopharyngeal carcinoma, is very rare. The current study reported an elderly woman with nasopharyngeal carcinoma who had recurrent syncopal attacks as the initial presentation, suggesting that great importance should be attached to the rare causes of syncope especially among patients with nasopharyngeal carcinoma, since it is an insidious malignancy which needs to be precisely identified.

The informed consent was obtained from the patient. All the personal information has been anonymized before the analysis.

## Case Report

A 66-year-old woman presented with a history of frequent and recurrent syncope which had lasted for half a month, and the frequency was up to 2 to 4 times per week. She was suffering from palpitation, dizziness, and neck tightness before the attacks, then the loss of consciousness followed by those symptoms. The syncopal episodes lasted about 30–60 Sec each time. Recovery would spontaneously occur with a complaint of weakness. Past medical history was only remarkable for hypertension and her parents both had coronary atherosclerotic heart disease.

On admission, she was in sinus rhythm with a heart rate of 74 beats/min and her blood pressure was 125/74 mmHg. Physical and neurological examination showed no obviously abnormal signs. The result of carotid sinus massage was negative. Electrocardiogram (ECG) indicated sinus bradycardia (58 beats/min). The color sonography cardiac ultrasound result was normal for the 66-year-old female. The 24-h ECG Holter monitor detected that the average heart rate was more than 60 beats/min, that the lowest heart rate was 38 beats/min, and that the longest RR interval was 1.73 Sec. There were no abnormalities in routine blood examination, electrolytes, glucose, liver and kidney function. The level of adrenocorticotropic hormone and catecholamine in plasma was normal. Similarly, electroencephalogram (EEG) was negative as computed tomography (CT) and angiography of the carotid and cerebral arteries were conducted.

**Figure 1:**
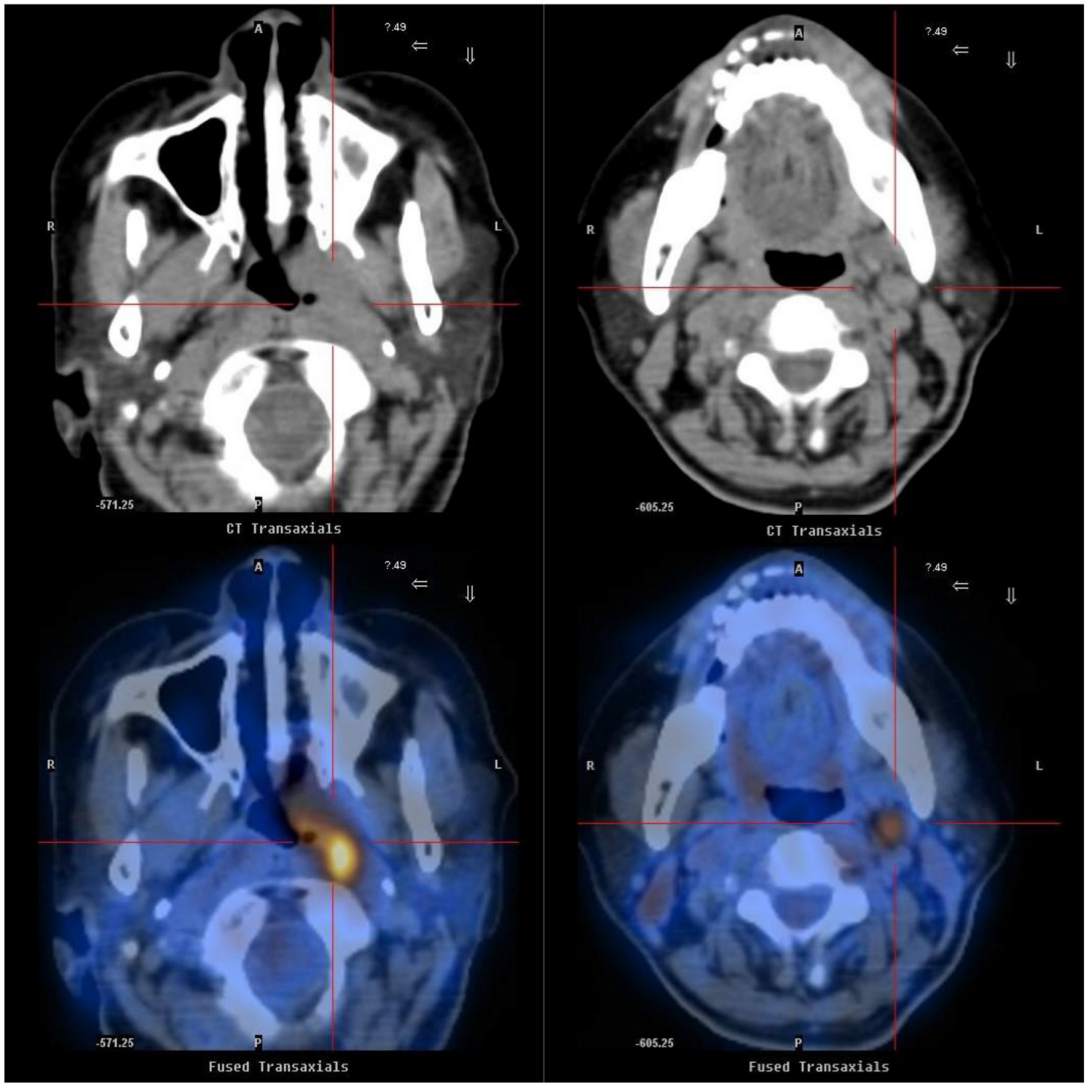
Positron emission tomography (PET) scan shows left nasopharyngeal carcinoma extending to carotid sinus. PET scan demonstrated an increased accumulation of FDG (2-deoxy-2-[18F] fluoro-D-glucose) in the thickening left nasopharynx (left) and an increased accumulation of FDG in the left carotid sheath space (right).

During hospitalization, her bedside ECG indicated sinus arrest and ventricular escape rhythm when she experienced syncope. These symptoms were followed by the loss of consciousness with blood pressure of 63/34 mmHg and pulse of 39 beats/min. Whereafter, the patient received treatment of temporary pacemaker during hospital stay. Simultaneously, the coronary angiography demonstrated coronary atherosclerosis without significant stenosis. However, she still had syncopal attacks after pacemaker implantation while ECG suggested that pacing rhythm alternated with sinus rhythm of 60 beats/min and blood pressure of 59/39 mmHg. The abdominal ultrasonography showed no evident adrenal mass but surprisingly detected multiple low echo-level masses in her liver. The contrast-enhanced abdominal CT revealed multiple low-density shadows of varied sizes in the liver, which were diagnosed as secondary malignant tumors. Systemic metabolic imaging were performed to identify the primary tumor. The positron emission tomography (PET) scan demonstrated an increased accumulation of FDG (2-deoxy-2-[18F] fluoro-D-glucose) in the thickening left naso-pharyngeal mucous membranes and the left carotid sheath space (Figure 1). Nasopharyngeal biopsy revealed non-keratinized nasopharyngeal carcinoma. Hence, palliative chemoradiotherapy was administered after the diagnosis of advanced nasopharyngeal carcinoma.

Three months after diagnosis and treatment, the left nasopharyngeal and carotid sheath space masses were significantly reduced in size, and the patient had no recurrence of syncope in 2 years. Currently, the patient has been still receiving palliative treatment.

## Discussion

Syncope is defined as a self-limited transient loss of consciousness due to a global reduction in cerebral perfusion (1, 3). Generally speaking, several causes may contribute to syncope. Except for some unexplained pathogenesis, syncope can be categorized as cardiac syncope, reflex syncope, orthostatic hypotension and cerebral syncope. For the cardiac syncope, the primary mechanism is a remarkable reduction in cardiac output due to cardiopulmonary disease, such as arrhythmia, structural heart disease, or pulmonary embolism that contribute to global cerebral hypoperfusion (4). Thus, cardiac syncope may be preceded by palpitations, shortness of breath, or chest pain. For patients with arrhythmic syncope, a pacemaker therapy may be of benefits. In reflex syncope, peripheral vasodilation and bradycardia induced by abnormal sympathetic or vagal reflexes can lead to sudden drop in blood pressure and decrease in cardiac output (5). According to the current pathophysiological mechanisms, reflex syncope can be summarized as vasovagal syncope, carotid sinus syndrome, and urinary syncope. Orthostatic hypotension (OH) is an excessive fall in blood pressure after standing which may result from inadequate intravascular volume, autonomic nervous system dysfunction, decreased venous return, or an inability to increase cardiac output in response to postural changes. Transient loss of consciousness with spontaneous recovery, which has a direct impact on cerebral parenchyma or cerebral vessels, can also be ascribed to cerebral syncope like epilepsy, transient ischemic attack (TIA) or blood supply disorders of vertebrobasilar arteries.

The current study reported a patient with carotid sinus syndrome accompanied by arrhythmia. In this case, the patient was an elderly female with presentation of cardiovascular disease like palpitation, pallor and syncope. The ECG indicated sinus arrest and ventricular escape rhythm during a syncopal attack. In addition, she had a past medical history of hypertension and a family history of cardiovascular disease, suggesting possibility of cardiac syncope. From another perspective, The PET scan demonstrated a lesion of carotid sheath space, secondary to advanced nasopharyngeal carcinoma, anatomically indicative of carotid sinus syndrome. Notably, our patient still had syncopal attacks and hypotension after the pacemaker therapy, which implied that cardiac syncope might be precluded. Therefore, it could be inferred that her syncope was related to carotid sinus syndrome.

Carotid sinus syndrome (CSS) is a clinical syndrome which presents as a group of symptoms including spontaneously sudden onset of dizziness, weakness, tinnitus or even syncope. The associated symptoms directly reflect carotid sinus hypersensitivity (CSH) due to compression or invasion of the carotid sinus. Four pathophysiological mechanisms of carotid sinus syndrome have been described (6): the cardio-inhibitory type, the vasodepressor type, the mixed type and the rare cerebral type. Under normal circumstances, a pacemaker therapy might alleviate syncope originated from the cardio-inhibitory type of CSS. However, the pacing might be ineffective if pure vasodepressor syncope develops. In this case, since the patient still had recurrences of syncope and hypotension after pacemaker implantation, the vasodepressor or mixed type of carotid sinus syndrome was probably the main pathogenesis.

In the setting of head and neck cancer, the causative mechanisms of syncope include irritation of carotid sinus or glossopharyngeal nerve (7). In this case, our patient was an elderly female ever diagnosed with nasopharyngeal carcinoma, who had syncopal episodes as the initial symptom. The PET scan indicated a diagnosis of advanced nasopharyngeal carcinoma with involvement in carotid sheath space, anatomically supporting the possibility of compression on the carotid sinus. It can be deduced that, recurrent syncopal attacks were associated with carotid sinus syndrome, secondary to mechanical compression and stimulation of the carotid sinus, resulting from the metastasis of advanced nasopharyngeal carcinoma. Accordingly, the exceptional causes of syncope should be taken into account, especially in the case of head and neck cancer like nasopharyngeal carcinoma.

The cases of syncope associated with nasopharyngeal carcinoma reported between 1990 and 2020 have been summarized (Table 1). The majority of nasopharyngeal carcinoma patients (86.36%) with syncope were males, indicating that male patients with nasopharyngeal carcinoma may be prone to developing syncope. Moreover, patients with syncopal attacks mostly have a late or advanced state of illness. According to the previous studies, most patients were diagnosed as clinical III stages, whose biopsy identifying low differentiated /undifferentiated squamous cell carcinoma. It is indicated that syncope may be associated with the metastasis of advanced nasopharyngeal carcinoma. Meanwhile, approximately one fifth of patients with nasopharyngeal carcinoma died consequently. Thus, syncope appears to be a poor prognostic sign of tumor invasion and metastasis. In addition, carotid sinus syndrome is found to be responsible for most syncope caused by nasopharyngeal carcinoma, which is primarily attributed to the compression of metastatic mass lesions on carotid sinus.

**Table 1:** Summary of the cases of syncope associated with nasopharyngeal carcinoma.

**Article**	**Age/gender**	**Time**	**Stages/typing**	**ECG**	**Treatment**	**Outcome**	**Cause**	**Possible etiology**
Degirmenci (8)	56/M	NA	III/Low differentiated squamous cell carcinoma	Sinus bradycardia	Chemoradiotherapy, lymphadenectomy	No recurrence	Metastatic mass lesions compressing	CSS
Wang (9)	75/M	2 y	III/Low differentiated squamous cell carcinoma	Third degree A-V block	Chemoradiotherapy	No recurrence	Metastatic mass lesions compressing	CSS
Zhang (10)	75/M	2 m	III/NA	sinus arrest, sick sinus syndrome	Chemoradiotherapy	NA	Downregulation of NE secretion	CSS/CSH
Lin (11)	72/M	5 m	IV/Non-keratinizing squamous cell carcinoma	Normal	Radiotherapy	Death	Involvement of glossopharyngeal and vagus nerves	PSL
	66/M	5 m	IV/Undifferentiated squamous cell carcinoma	Normal	Chemoradiotherapy	NA	Involvement of glossopharyngeal and vagus nerves	PSL
Kala (12)	69/M	5 y	III/Low differentiated squamous cell carcinoma	Normal	Chemoradiotherapy	No recurrence	Irritation of vagus nerve	Irritation of vagus nerve
Atsuumi (13)	53/M	9 y	III/Low differentiated squamous cell carcinoma	NA	Chemotherapy	No recurrence	Irritation of vagus nerve	PSL
Tang (14)	54/M	NA	IV/Undifferentiated squamous cell carcinoma	Normal	Chemoradiotherapy	No recurrence	NA	CSS
	65/M	NA	IV/Undifferentiated squamous cell carcinoma	Sinus bradycardia	Chemoradiotherapy	No recurrence	NA	Glossopharyngeal neuralgia
	57/M	NA	IV/Undifferentiated squamous cell carcinoma	Normal	Chemoradiotherapy	No recurrence	NA	PSL
Chen-Scarabelli (7)	62/M	6 m	IV/Keratinizing squamous cell carcinoma	Bradycardia, long sinus pauses	Chemoradiotherapy	No recurrence	Metastatic mass lesions compressing	CSS/CSH
Wang (15)	50/M	6 m	III/Undifferentiated squamous cell carcinoma	Sinus arrest	Chemoradiotherapy	No recurrence	Metastatic mass lesions compressing	CSS, PSL, GNI
Tulchinsky (16); Wang (17); Li (18)	68/M 55/F 65/M	3 m NA 10 d	IV/Low differentiated squamous cell carcinoma III/ Undifferentiated non-keratinizing carcinoma III/ Undifferentiated non-keratinizing carcinoma	Junctional rhythm, ventricular escape NA Normal	Chemoradiotherapy Chemoradiotherapy Chemotherapy	Death No recurrence No recurrence	Metastatic mass lesions compressing Metastatic mass lesions compressing Metastatic mass lesions compressing	CSS CSS CSS
Zhang (19)	53/M 64/M	2 d 2 y	III/NA III/ Non-keratinizing squamous cell carcinoma	Atrial fibrillation bradycardia	Chemoradiotherapy Radiotherapy	No recurrence Recurrence	Irritation of vagus nerve Metastatic mass lesions compressing	PSL CSS
Yang (20)	56/M	7 y	III/Low differentiated squamous cell carcinoma	Third degree A-V block	Radiotherapy	Death	NA	CSS
	36/F	1 y	III/Low differentiated squamous cell carcinoma	Third degree A-V block	Radiotherapy	Death	NA	CSS
Zhou (21)	48/M	5 y	III/Low differentiated squamous cell carcinoma	Sinus bradycardia	Radiotherapy	NA	NA	CSS, GNI
Shen (22)	63/M	1 y	III/Low differentiated squamous cell carcinoma	Sinus bradycardia	Radiotherapy	No recurrence	NA	CSS
Our case	66/F	2 y	IV/Non-keratinizing squamous cell carcinoma	Third degree A-V block	Chemoradiotherapy	No recurrence	Metastatic mass lesions compressing	CSS

*M, male; F, female; y, years; m,months; d, days; NA, not available; ECG, electrocardiogram; CSS, carotid sinus syndrome; PSL, parapharyngeal space lesions; GNI, glossopharyngeal nerve injury. Time: Period from time of syncope occurrence to time of diagnosis*.

The patient in this case was eventually diagnosed with advanced nasopharyngeal carcinoma. However, she did not suffer from typical symptoms like blood tinged sputum, nosebleed, hearing loss or headache. In contrast, her initial presentation was syncope associated with carotid sinus syndrome. Most physicians may neglect nasopharyngeal malignancies characterized by syncope and hypotension. It is recommended that doctors should evaluate the origin of syncope before the pacemaker treatment. Although a pacemaker can maintain a normal heart rate, the efficacy is limited when mentioning the vasodepressor or mixed type of carotid sinus syndrome.

To summarize, this was the first case of advanced nasopharyngeal carcinoma with recurrent syncope as an initial symptom. To our knowledge, syncope rarely occurred as the initial presentation of advanced nasopharyngeal carcinoma, and carotid sinus syndrome was considered as the potential intrinsic cause of syncope in nasopharyngeal carcinoma (7–10, 14–22). Hence, cranial imaging examinations and nasopharyngoscopy may be needed for these suspected patients. Chemoradiotherapy could be a better choice for controlling syncope (7–9, 12, 14, 15, 17, 19, 23). It is also advised that physicians should find out the origin of recurrent syncope through clinical condition and relevant tests, in the purpose of early diagnosis, precise treatment and improved prognosis.

## Data Availability Statement

The raw data supporting the conclusions of this article will be made available by the authors, without undue reservation.

## Ethics Statement

Written informed consent was obtained from the individual(s) for the publication of any potentially identifiable images or data included in this article.

## Author Contributions

Y-SG, S-CZ, and M-QL: case collection. Y-SG, S-CZ, M-QL, L-WZ, and K-YL: analysis of the case. S-CZ and M-QL: draft manuscript preparation. All authors reviewed the results and approved the final version of the manuscript.

## Funding

This study was funded by a grant from National Natural Science Foundation of China General Program (Grant Number: 81873495), Heart Failure Center Research Fund of Fujian Provincial Hospital (supported by Fujian Provincial Department of Finance), Natural Science Foundation of Fujian Province (Grant Number: 2018J01242). The grants played a role in the design of the study, collection of data, follow-up of the patients, interpretation of data and in writing the manuscript.

## Conflict of Interest

The authors declare that the research was conducted in the absence of any commercial or financial relationships that could be construed as a potential conflict of interest.

## Publisher's Note

All claims expressed in this article are solely those of the authors and do not necessarily represent those of their affiliated organizations, or those of the publisher, the editors and the reviewers. Any product that may be evaluated in this article, or claim that may be made by its manufacturer, is not guaranteed or endorsed by the publisher.
